# Trypanosomatidae (Kinetoplastea: Trypanosomatida) diversity infecting the South American coati *Nasua nasua* (Carnivora: Procyonidae) in urban forest fragments in the Brazilian Midwest

**DOI:** 10.3389/fcimb.2026.1822974

**Published:** 2026-04-22

**Authors:** Gabriel Carvalho de Macedo, Wanessa Teixeira Gomes Barreto, Carina Elisei de Oliveira, Leonardo França do Nascimento, Andreza Castro Rucco, Julia Gindri Bragato Pistori, William Oliveira de Assis, Geovanna Silva dos Santos, Wesley Arruda Gimenes Nantes, Gisele Braziliano de Andrade, André Luiz Rodrigues Roque, Heitor Miraglia Herrera

**Affiliations:** 1 Laboratory of Trypanosomatid Biology, Oswaldo Cruz Foundation, Rio de Janeiro, Rio de Janeiro, Brazil; 2 Department of Environmental Sciences and Agricultural Sustainability, Dom Bosco Catholic University, Campo Grande, Mato Grosso do Sul, Brazil; 3 Department of Ecology and Conservation, Federal University of Mato Grosso do Sul, Campo Grande, Mato Grosso do Sul, Brazil; 4 Laboratory of Spatial Ecology and Conservation, São Paulo State University, Rio Claro, São Paulo, Brazil; 5 Department of Biotechnology, Dom Bosco Catholic University, Campo Grande, Mato Grosso do Sul, Brazil

**Keywords:** Carnivora, key-host species, *Leishmania amazonensis*, *Leishmania infantum*, SSU rRNA, *Trypanosoma cruzi*, urban environment, wild mammal species

## Abstract

**Introduction:**

South American coatis are reported as key-hosts of trypanosomatid species in Brazilian Pantanal, presenting high parasitemia and infection rates, long-lasting infections, ability to bioaccumulate *Trypanosoma evansi*, *T. rangeli* and different genotypes of *T. cruzi*, and biological features that favor these parasites’ transmission. Moreover, this mammal species was also reported parasitized by the monoxenous trypanosomatid *Crithidia mellificae* and included in the epidemiological scenario of Visceral Leishmaniasis in Campo Grande (CG), Brazilian Midwest. Within this context, the aim of this work was to assess the diversity of trypanosomatid species in coatis from CG.

**Methods:**

Samples of 110 South American coatis captured in two forest fragments were submitted to Nested Polymerase Chain Reaction (nPCR) of small ribosomal ribonucleic acid subunit (SSU rRNA) gene and Sanger sequencing analyses.

**Results:**

The positivity rate of nPCR was 36.4% (40/110) among individuals, including infections by *Leishmania infantum*, *L. amazonensis*, *T. cruzi* and the Molecular Operational Taxonomic Unit Trypanosomatidae sp. CROT. When combining SSU rRNA detection and sequencing with previous published data from the same coati cohort, we observed a positivity rate of 47.3% (52/110), in single (36.4%; n = 40/110) or mixed Trypanosomatidae infections (10.9%; n = 12/110).

**Discussion:**

Our findings indicate the South American coati is a key-hosts species in the ecology of trypanosomatids also in the urban environment of CG.

## 1 Introduction

Trypanosomatids (Kinetoplastea; Trypanosomatida) comprise unicellular flagellate parasites with marked differences in their survival strategies and life cycles. Trypanosomatidae includes both insect monoxenous parasites, which comprise around 24 genera such as *Leptomonas*, *Herpetomonas*, *Crithidia*, *Sergeia*, living their entire life cycle in one invertebrate host, and dixenous parasites, which are capable to switch between an invertebrate and a vertebrate host (*Trypanosoma*, *Leishmania*, *Endotrypanum*, and *Porcisia*), or an invertebrate and a plant (*Phytomonas*). Despite the broad spectrum of genera within this family, most scientific studies focus on pathogenic species responsible for important diseases in humans and domestic animals (Lukeš et al., 2018; Kostygov et al., 2021; Lukeš et al., 2021). The recent application of new molecular tools with higher analytical capabilities has been increasing new trypanosomatid species as *Trypanosoma* sp (Cottontail et al., 2014) and molecular operational taxonomic units (MOTUs) as *Trypanosoma* sp. DID (Rodrigues et al., 2019; Nantes et al., 2021; Dario et al., 2022; Júnior et al., 2025), *Trypanosoma* sp. Neobats (Alves et al., 2021), Trypanosomatidae sp. ‘*strain EVA’* (Svobodová et al., 2007) and Trypanosomatidae sp. CROT (Nantes et al., 2024). This context demonstrates that the ecology of Trypanosomatidae remain poorly elucidated, with many unanswered questions regarding life cycle, diversity, and phylogenetic relatedness between clades (Rodrigues et al., 2019).

In natural landscapes, often characterized by high biodiversity, these parasites established relationships with different mammalian host species that play distinct roles in their transmission cycles and constitute reservoir systems (Herrera et al., 2004, 2005; Roque and Jansen, 2014; Morales et al., 2017; Jansen et al., 2018). These relationships are characterized by chronic and less pathogenic infections, high ecological specificity and mutual adaptation between trypanosomatids and their hosts, which contribute for maintaining an ecological stability in the transmission cycles (Jansen and Roque, 2010; Jansen et al., 2018). On the other hand, urban and peri-urban environments suffer continuous anthropogenic pressures, characterized by forest disturbance and fragmentation, habitat and biodiversity loss, changes in blood meal availability for vectors, and unpredictable variations in the transmission cycles (Calegaro-Marques and Amato, 2014; de Souza and Weaver, 2024). These ecological shifts may favor spillover events by modifying the local enzooty and including new host populations that are not usually considered in reservoir systems. This is exemplified by the occurrence of *Crithidia* spp. and *T. janseni* in bats (Rangel et al., 2019; Torres et al., 2024), mixed infections of *Leishmania* with *Crithidia* and *Leptomonas* in humans (Maruyama et al., 2019; Thakur et al., 2020; Rogerio et al., 2023), atypical human trypanosomiasis due to *T. evansi* and *T. lewisi* (Truc et al., 2013; Chau et al., 2016; Jain et al., 2023), *T. lainsoni* infection in opossums (Nantes et al., 2021), and *T. cruzi* and *L. infantum* infections in viperid snakes (Nantes et al., 2024).

The South American Coati (*Nasua nasua*) (hereafter “coati”) is a carnivorous species from the Procyonidae family with a well-established role in the life cycle of some trypanosomatid species in the Brazilian Pantanal, the world`s largest tropical wetland. This mammal species presented high prevalences of infection, high parasitemia rates and mixed infections by *T. cruzi*, *T. evansi* and *T. rangeli* (Herrera et al., 2004; Alves et al., 2011; Herrera et al., 2011; Rocha et al., 2013; Alves et al., 2016; Santos et al., 2019). Bioaccumulation of different *T. cruzi* DTUs was reported (Rocha et al., 2013), with infections by TcI and TcII persisting for up to eight months in the same individuals (Herrera et al., 2008). Also, season, age and sex are predictive factors in the *T. cruzi* and *T. evansi* transmission among coatis from Pantanal: higher parasitemia rates of *T. cruzi* were observed in females during the dry season, and higher prevalence and parasitemia rates of *T. evansi* were observed in adult individuals (Alves et al., 2011).

Some biological features of this mammal species support its role in the maintenance and transmission of these parasites, especially in the case of *T. cruzi*. Coatis can interact indirectly with other hosts in nature through their arboreal nests built for resting and reproduction (Gompper and Decker, 1998), which can be frequented by other mammal and invertebrate species (de Lima et al., 2015; Santos et al., 2015; Monticelli and Gasco, 2018). In Pantanal region, Alves et al. (2016) demonstrated a high detection rate of *T. cruzi* in *Triatoma* sp. and *Rhodnius* sp. collected from four coati’s arboreal nests. Pessanha et al. (2023) reported a high detection rate of TcI and TcII in 23 *T. sordida* caught from a single nest and described through genetic analyses that the collared anteater (*Tamandua tetradactyla*) was the blood meal source of these triatomines. While contaminative route (metacyclic forms expelled in triatomine feces) may be frequent in the coati’s nests (Jansen et al., 2018), the coati’s omnivorous diet, which include insects and small mammals (Alves-Costa et al., 2004; Bianchi et al., 2014), certainly assure oral transmission, described as the most probable main *T. cruzi* dispersion strategy in nature (Jansen et al., 2018). Additionally, the fact that coatis are scansorial animals reinforces that they can contribute to the transition of transmission cycles from one extract to another.

Coatis are well-adapted to urban environments due to the absence of natural predators and the large supply of food discarded by humans in can contents, which favor their reproducibility and their population stability (Alves-Costa et al., 2004; Estevam et al., 2020). In Campo Grande (CG), Brazilian Midwest, a municipality that shelter around 100,000 hectares of vegetation cover distributed in parks, squares and conservation units (Oppliger et al., 2019), coati populations thrive with high-density populations (Costa et al., 2009; Barreto et al., 2021). In this region this species displayed high rates of *L. infantum* infection, presence of DNA in intact skin and ability to sustain viable parasites in bone marrow, and *C. mellificae* was isolated from blood of six individuals (Dario et al., 2021; de Macedo et al., 2023). Furthermore, several trypanosomatid species such as *Trypanosoma cruzi*, *T. cruzi marinkellei*, *T. janseni*, *T. lainsoni*, *T. minasense*, *T. rangeli*, *T. dionisii*, *Trypanosoma* sp. DID, Trypanosomatidae sp. CROT, *L. amazonensis* and *L. braziliensis* have been reported in many wildlife hosts from forested fragments of CG (Castro et al., 2020; Nantes et al., 2021 and 2024; Torres et al., 2024; Junior et al., 2025). Within this context, we aimed to access the diversity of trypanosomatid species in coatis from CG through the molecular detection of the small subunit ribosomal ribonucleic acid (SSU rRNA) gene followed by DNA sequencing analysis.

## 2 Materials and methods

### 2.1 Origin of samples

In this study we explored samples of blood and bone marrow (BM) of 110 coatis previously investigated. We combined our results with data of biphasic medium isolation obtained by Dario et al. (2021), and Heat-Shock-Protein 70 (hsp70) sequencing, Kinetoplast DNA (kDNA) detection, and biphasic medium isolation obtained by de Macedo et al. (2023). Three hundred and twenty-two samples (190 blood samples and 132 BM samples) from two areas (Prosa State Park [PSP] [20°26’59’’S, 54°33’55’’O] and Air Force Village [AFV] [20°28’17’’S, 54°39’14’’O]) were included in this study. The description of the study areas, the proceedings of capture and recapture of the animals, and the collection and storage of the samples are described by de Macedo et al. (2023). All the field procedures were performed according to the Instituto Chico Mendes de Conservação da Biodiversidade (ICMBIO) (license number 56912-2), the Instituto de Meio Ambiente de Mato Grosso do Sul (IMASUL) (license number 05/2017, process No.61/405959/2016), an Air force cooperation agreement (No.01/GAP-CG/2018), and the Ethics Committee for Animal Use of Universidade Católica Dom Bosco (UCDB), CG, MS (license number 001/2017).

### 2.2 Detection of trypanosomatid DNA

Genomic DNA was extracted from 200 µL of whole blood and bone marrow samples using DNeasy Blood and Tissue Kits (Qiagen^®^, Germany). The DNA was eluted in 100 µL elution buffer and stored at -20 °C until molecular analysis. The concentration and quality were assessed by spectrophotometry (Biodrop, Analítica ^®^, Brazil). All samples were screened in a conventional Polymerase Chain Reaction (cPCR) of the ß-globin gene as described by Greer et al. (1991) to verify the viability of DNA samples. Molecular detection of Trypanosomatidae was performed through Nested PCR (nPCR) targeting the small ribosomal ribonucleic acid subunit (SSU rRNA) according to Smith et al. (2008). The PCR products were visualized on 1.5% agarose (Kasvi ^®^, Brazil), stained with Gel Red Nucleic Acid Stain (Biotium ^®^, EUA).

### 2.3 Sequencing and phylogenetic analysis

The nPCR amplified DNA samples were purified through the ExoSAP-IT PCR Product Cleanup Reagent (Applied Biosystems ^®^, United States) and sequenced in both directions in AB3500 platform (Applied Biosystems ^®^, United States) using the 3.1 Big Dye Terminator kit (Applied Biosystems ^®^, United States). Consensus sequences were generated and compared through BLAST analysis (https://blast.ncbi.nlm.nih.gov/Blast.cgi), and the cut-off values for species determination were 100% query coverage, 99% genetic identity and E-value equal to zero.

The sequences were edited and aligned in the Geneious Prime software (https://www.geneious.com) using the MUSCLE algorithm with the default parameters described by Edgar (2004). The most suitable substitution model for each analysis was chosen with the Bayesian Information Criterion (BIC) using jModelTest (Darriba et al., 2012). In the BEAST program (Bouckaert et al., 2019), we performed ten independent runs of 2x10^7^, sampling every 2x10^3^ generation. The parameters were considered adequate by TRACER v.1.7.2 (Rambaut et al., 2018) where the effective sample size (ESS) values were greater than 200. In addition, a burn-in of 10% of each LogCombiner run was applied. The final tree was generated with Maximum Clade Credibility (MCC) after a 10% burn-in using Tree Annotator. The reconstruction of the Bayesian inference tree was visualized in FigTree v.1.4.3 (Rambaut, 2016).

### 2.4 Parasitological detection of Trypanosomatidae

Because positive culture of trypanosomatids indicate high parasitemia and higher transmission potential (Alves et al., 2016), we attempted to isolate trypanosomatids from the sampled material in PSP by inoculating approximately 0.3 ml of whole blood samples in Neal, Novy, Nicolle (NNN) + Lit biphasic medium supplemented with fetal bovine serum (10%). When the cultures reached exponential phase of growth, the samples were submitted to DNA extraction and deposited in Wild, Domestic Mammals and Vectors Collection of Trypanosoma (FIOCRUZ – COLTRYP). The sampled material from AFV was previously analyzed and published in Dario et al. (2021).

## 3 Results

### 3.1 Sequences obtained through SSU rRNA nPCR and positive rates

Trypanosomatid DNA was amplified in 69 samples (46 blood and 23 BM) of 44 coatis, representing 40% (44/110) of SSU rRNA amplification among individuals. There were 33 positive samples (24 blood and nine BM) of 23 individuals from PSP, and 36 positive samples (22 blood and 14 BM) of 21 individuals from AFV. Consensus sequences presenting query coverage and genetic identity within the cut-offs were generated from 50 samples of 32 individuals and were deposited in GenBank. Among these sequences, we detected *L. infantum* (n=33 samples), *L. amazonensis* (n=10 samples), *T. cruzi* (n=6 samples), and one Trypanosomatidae sp. CROT. Another sequence (UCDB 411D) presented 98.85% of genetic identity and was characterized as *L. amazonensis* through phylogenetic analysis (**Table 1**).

**Table 1:** Description of DNA sequences obtained through SSU rRNA nPCR in blood and bone marrow samples of South American coatis (*Nasua nasua*) captured in two forest fragments of Campo Grande, Midwest Brazil, from March 2018 to May 2019.

Tripanosomatid species	Animal ID	Area	GenBank accession number	Sample type	Query coverage	Genetic identity	GenBank homologous sequence
Trypanosomatidae CROT	UCDB 418B	PSP	PP721003	B	100%	100%	OR371592
*Trypanosoma cruzi*	UCDB 332	PSP	PP719872	B	100%	99.54%	AY785573
	UCDB 335	PSP	PP719871	B	100%	100%	AY785573
	UCDB 345	AFV	PP719869	B	100%	100%	MT994566
	UCDB 357B	PSP	PP719868	B	100%	100%	PP935715
	UCDB 397	PSP	PP719874	BM	100%	99.83%	OQ418061
	UCDB 411B	AFV	PP719867	B	100%	100%	AJ009148
*Leishmania amazonensis*	UCDB 324	PSP	PP718783	BM	100%	100%	X53912.1
	UCDB 324D	PSP	PP718784	BM	100%	99.63%	X53912.1
	UCDB 328C	PSP	PP718782	BM	100%	100%	X53912.1
	UCDB 338	PSP	PP718785	BM	100%	100%	X53912.1
	UCDB 357	PSP	PP718779	B	100%	99.77%	X53912.1
	UCDB 411C	AFV	PX070518	B	100%	100%	X53912.2
	UCDB 411D*	AFV	PX609968	B	100%	98.85%	X53912.1
	UCDB 411G	AFV	PP718777	B	100%	100%	X53912.1
	UCDB 415	PSP	PP718776	B	100%	100%	X53912.1
	UCDB 517C	AFV	PP718781	BM	100%	100%	X53912.1
	UCDB 518B	AFV	PP718780	BM	100%	99.82%	X53912.1
*Leishmania infantum*	UCDB 324C	PSP	PX609966	B	100%	99.22%	XR_001203206.1
	UCDB 325	PSP	PP725754	B	100%	100%	XR_001203206.1
	UCDB 325B	PSP	PX609967	B	100%	99.81%	XR_001203206.1
	UCDB 326B	PSP	PP725752	B	100%	99.82%	XR_001203206.1
	UCDB 327	PSP	PP725751	B	100%	100%	XR_001203206.1
	UCDB 327C	PSP	PP725750	B	100%	100%	XR_001203206.1
	UCDB 331	PSP	PP735724	B	100%	100%	XR_001203206.1
	UCDB 332	PSP	PP725766	BM	100%	100%	XR_001203206.1
	UCDB 333	PSP	PP735723	B	100%	100%	XR_001203206.1
	UCDB 333	PSP	PP725765	BM	100%	100%	XR_001203206.1
	UCDB 334	PSP	PP735722	B	100%	99.60%	XR_001203206.1
	UCDB 334	PSP	PP725767	BM	100%	99.60%	XR_001203206.1
	UCDB 336	PSP	PP735721	B	100%	99.10%	XR_001203206.1
	UCDB 349	AFV	PP725764	BM	100%	100%	XR_001203206.1
	UCDB 349B	AFV	PP725763	BM	100%	100%	XR_001203206.1
	UCDB 352B	AFV	PP725762	BM	100%	100%	OR250394
	UCDB 352B	AFV	PP735719	B	100%	100%	OR250394
	UCDB 352C	AFV	PP725760	BM	100%	100%	OR250394
	UCDB 352C	AFV	PP725761	B	100%	100%	OR250395
	UCDB 353B	AFV	PP735718	B	100%	99.82%	XR_001203206.1
	UCDB 356	PSP	PP735717	B	100%	100%	OR250395
	UCDB 397B	PSP	PP735716	B	100%	99.82%	XR_001203206.1
	UCDB 401	AFV	PX070515	B	100%	100%	KU948458
	UCDB 401B	AFV	PP735715	B	100%	100%	XR_001203206.1
	UCDB 403B	AFV	PX070516	B	100%	99.83%	XR_001203206.1
	UCDB 407G	AFV	PX070517	B	100%	100%	XR_001203206.1
	UCDB 407G	AFV	PP725759	BM	100%	100%	XR_001203206.1
	UCDB 410	AFV	PP735714	B	100%	100%	XR_001203206.1
	UCDB 417B	PSP	PP735713	B	100%	100%	XR_001203206.1
	UCDB 513	AFV	PX070519	BM	100%	100%	XR_001203206.1
	UCDB 513	AFV	PX070520	B	100%	100%	XR_001203206.1
	UCDB 514	AFV	PP725757	BM	100%	100%	XR_001203206.1
	UCDB 516	PSP	PP725756	BM	100%	100%	XR_001203206.1

Animal ID – The letters after the numbers represent the recaptures. Area – AFV, Air Force Village; PSP, Prosa State Park. Sample type – B, Blood; BM, Bone Marrow. *Characterization was confirmed by phylogenetic analysis.

Ten samples (seven blood and three BM) of ten different individuals (UCDBs 328, 330, 344E, 346D, 352, 399, 409, 423, 503 and 547) did not generate consensus sequences. When comparing their sense and antisense strands in BLAST, they presented genetic identity to trypanosomatid species that varied from 75.7% to 99.48% with query coverage ranging from 74% to 99%. Therefore, these samples were considered as non-characterized trypanosomatids (NCT).

Eight samples (six blood and two BM) of eight different individuals (UCDBs 346, 349B, 355, 407D, 409B, 508C, 512 and 546) did not generate sequences and therefore were considered as negative for Trypanosomatidae infection, although positive in the SSU rRNA nPCR. Among these, four samples corresponded to individuals that had trypanosomatid detection (including NCT) in different capture events or in another type of sample (blood or BM) (UCDBs 346, 349, 407 and 409). In this sense, the positivity rate of SSU rRNA nPCR was 36.4% (40/110) among individuals, including two that were characterized as NCT and *L infantum* or *L. amazonensis* in two capture events.

One sequence recovered from a blood sample of the individual UCDB 418 presented 100% identity with Trypanosomatid DNA obtained from *Crotalus durissus* in CG/MS (OR371592), and nearly 98% of identity and query cover with the Trypanosomatidae sp. EVA strain sequence isolated from *Lutzomyia evansi* in Venezuela (AF071866).

The six *T. cruzi-*SSU rRNA sequences were obtained from five blood and one BM samples of different individuals, corresponding to 5.4% of sampled coatis (6/110). Three individuals presented the TcI in blood samples (UCDB 332, 335, and 357B), two individuals presented TcIV in blood samples (UCDB 345 and 411B), and only one presented the TcII/TcVI in the bone marrow (UCDB 397).

The DNA of *L. amazonensis* was detected in 11 samples (five blood and six BM) of 7.3% of sampled coatis (8/110). Two individuals presented positivity in more than one capture: the individual UCDB 324 presented positivity in BM samples in the first capture (March 2018) and in the fourth capture (October 2018), while the individual UCDB 411 had positive blood samples in the third (November 2018), fourth (January 2019) and seventh (November 2019) captures.

The 33 *L. infantum*-SSU rRNA sequences were obtained from 22 blood and 11 BM samples of 20% (22/110) of the sampled coatis. Eight SSU rRNA positive blood samples (UCDBs 325, 327, 333, 352B, 352C, 403B and 513), and seven SSU rRNA positive BM samples (UCDBs 333, 349B, 352B, 352C, 513, 514, and 516) had been previously demonstrated through BM hsp70 sequencing or kDNA detection (**Supplementary Material 1**). In contrast, eight BM samples previously confirmed through these methods did not present positivity at SSU rRNA nPCR, neither in blood nor BM (UCDBs 328B, 329, 330, 352, 353, 403, 412, and 420).

When combining SSU rRNA nPCR with BM hsp70 sequencing and kDNA detection, and biphasic medium isolation, we observed a *L. infantum* positivity rate of 25.4% (28/110) among individuals. We observed that six individuals presented positivity in more than one capture, ranging the capture interval between one and seven months. Nine individuals presented positivity in blood and BM at the same time of capture (UCDBs 325, 327, 333, 334, 352, 401, 403, 407, and 513) (**Supplementary Material 1**).

### 3.2 Mixed infections

Considering all employed diagnostic assays in different tissues and all capture events, we observed mixed trypanosomatid infections in 10.9% of captured coatis (12/110), and single infections in 36.4% (40/110). All together represents 47.3% (52/110) of positivity rate in PSP and AFV coati populations together.

Among the mixed infections, we highlight one case of a male individual from AFV (UCDB 411), which was infected by three trypanosomatid species. This individual was captured in seven occasions from June 2018 to November 2019, presented *T. cruzi* TcIV in the second capture (August 2018), *L. amazonensis* in the third (November 2018), fourth (January 2019) and last capture (November 2019) through SSU rRNA nPCR, and *C. mellificae* through NNN+Lit isolation in the fourth capture (**Supplementary Material 2**). The male individual UCDB 328 from PSP was captured three times from March to August 2018 and presented a NCT detection in blood during the first capture (March 2018), *L. infantum* detection through kDNA in BM during the second capture (May 2018), and *L. amazonensis* detection through SSU rRNA nPCR of BM in the last capture (August 2018). Although this NCT could indicate a different trypanosomatid from the species detected in the second and last captures, it was not confirmed in Sanger sequencing and therefore was not considered as a different one. In fact, NCT infection was not considered when an individual presented species confirmation in (i) other sample type, (ii) other capture, (iii) or presented confirmation of *C. mellificae* and/or *L. infantum* infection in the previous studies (UCDBs 328, 330, 344, 346, 352, 409 and 547).

**Figure 1:**
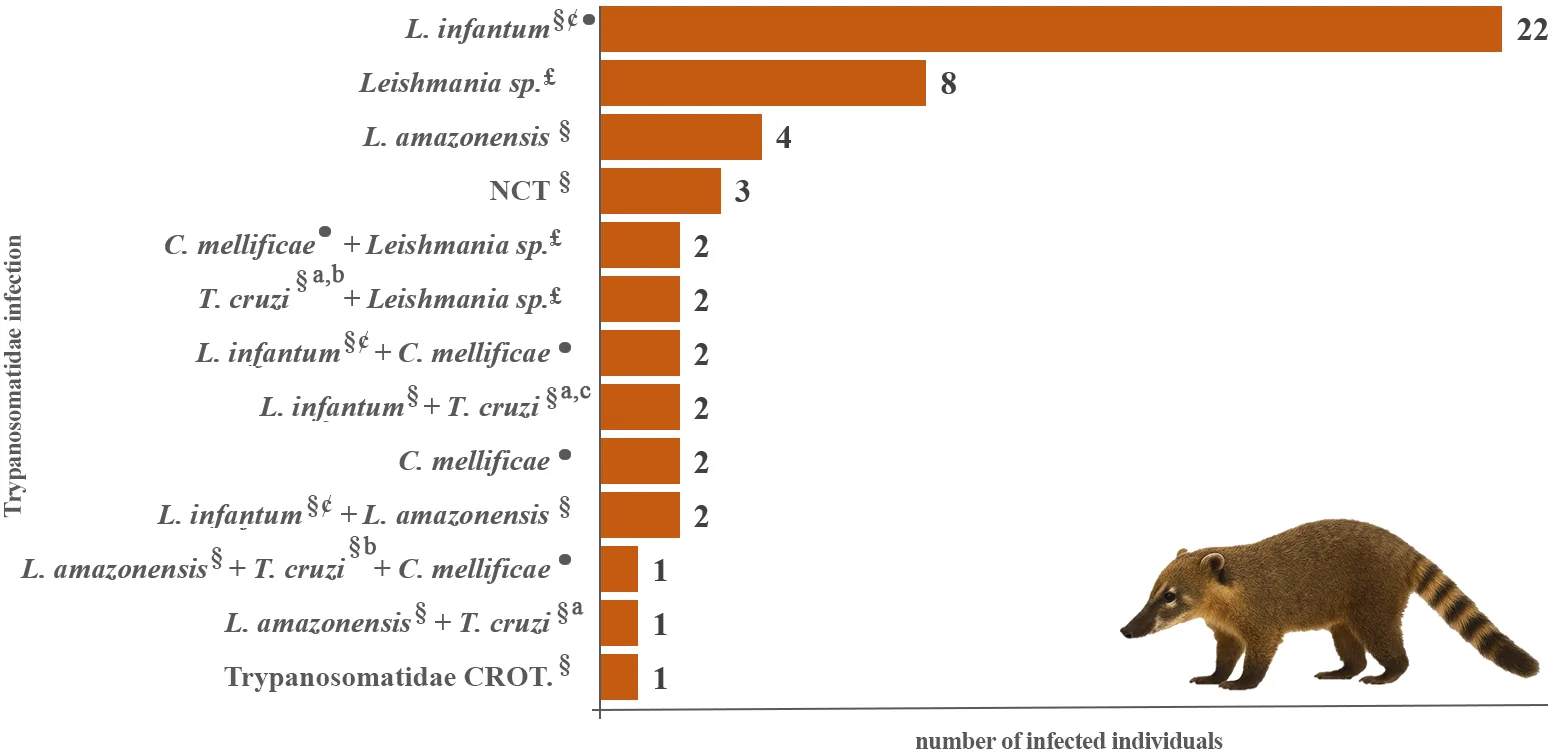
Single and mixed trypanosomatid infections in 52 South American coatis (*Nasua nasua*) sampled in two forest fragments of Campo Grande, Midwest Brazil, from March 2018 to May 2019. NCT, Non-characterized trypanosomatids. §: Infection determined by small subunit ribosomal ribonulcleic acid detection. ¢: Infection determined by heat-shock-protein 70 and *L. infantum* kinetoplast DNA by de Macedo et al. (2023). ●: Infection determined by isolation in biphasic media by Dario et al. (2021) and de Macedo et al. (2023). £: Infection determined by screening *Leishmania* spp. kinetoplast DNA detection by de Macedo et al., 2023. The upper letters indicate different *T. cruzi* Discrete Type Units: *a =* TcI; *b =* TcIV; *c =* TcII/TcVI. Trypanosomatidae CROT is a Molecular Operational Taxonomic Unit (MOTU) described by Nantes et al. (2024).

Interestingly, *T. cruzi* was detected in the sampled coatis only in mixed infections with *L. infantum* (UCDBs 332 and 397), *L. amazonensis* (UCDB 357), and *Leishmania* sp. (UCDB 335 and 345), besides UCDB 411. Co-infection by *L. amazonensis* and *L. infantum* was observed in two individuals from PSP (UCDBs 324 and 328). *C. mellificae* was also observed in mixed infections with *L. infantum* in two individuals (UCDB 403 and 407) and *Leishmania* sp. in other two individuals (UCDB 344 and 346), all of them in AFV (**Figure 1**).

### 3.3 Phylogenetic analysis

**Figure 2:**
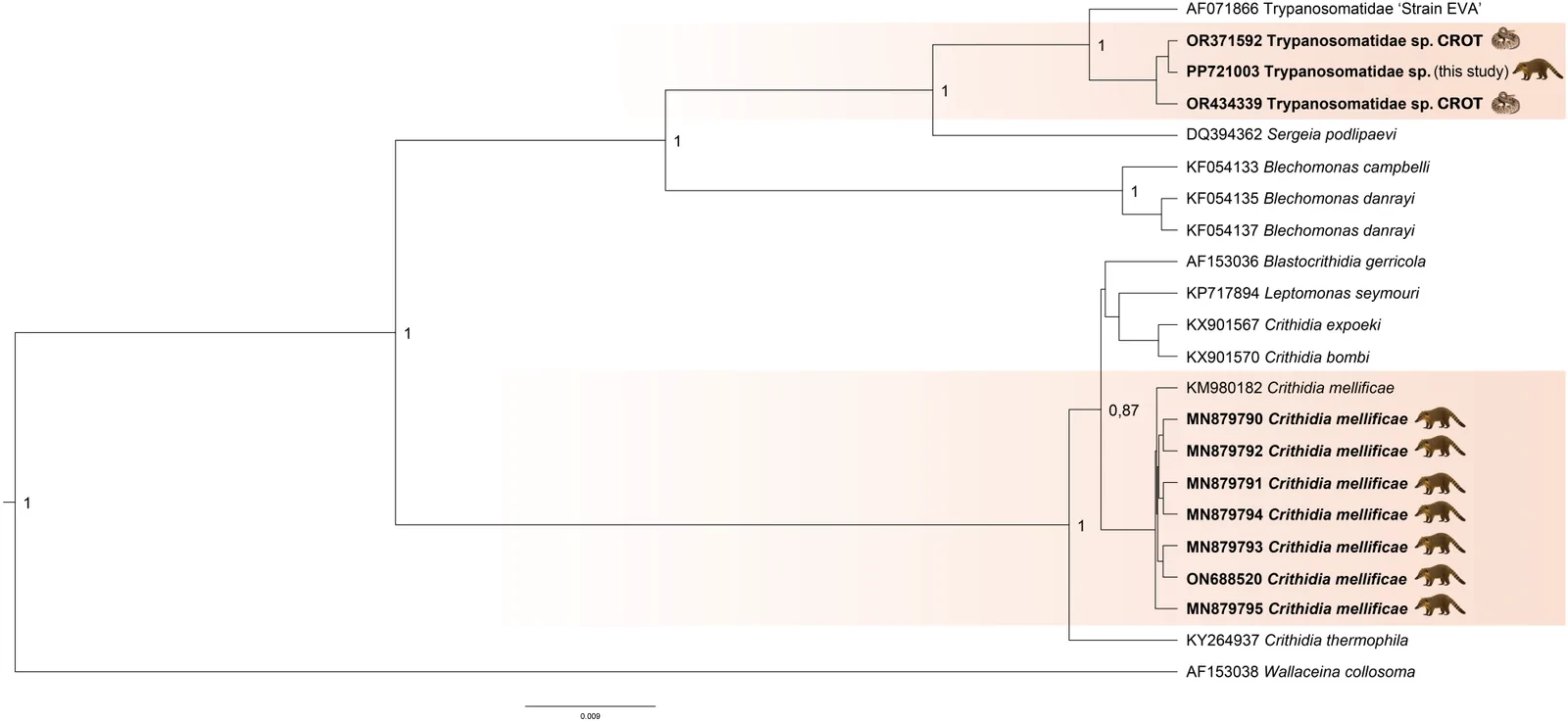
Bayesian inference of the Trypanosomatidae family based on the SSU rRNA gene isolated from different host species. The cladogram was inferred using the TrNef+G substitution model selected by the jmodelTest program. The species highlighted in bold are isolates found in the municipality of Campo Grande - MS. The species *Wallaceina collosoma* (AF153038) was used as the outgroup. Trypanosomatidae “Strain EVA” and Trypanosomatidae CROT are Molecular Operational Taxonomic Units (MOTUs) described by Svobodová et al. (2007) and Nantes et al. (2024), respectively.

Using a phylogenetic approach based on the SSU rRNA marker, we conducted three independent analyses. In the first analysis, which focused on selected members of the family Trypanosomatidae (**Figure 2**), we recovered a cohesive *Crithidia* clade that includes seven accessions of *C. mellificae* isolated from *N. nasua* within the municipality of CG, Mato Grosso do Sul. A second clade, composed of sequences identified as Trypanosomatidae CROT (OR371592, PP721003, and OR434339), was placed closer to the ‘Strain EVA’ lineage, whereas other reference genera—*Leptomonas seymouri*, *Blastocrithidia gerricola*, *Blechomonas campbelli/danrayi*, and *Sergeia podlipaevi*—formed distinct branches within the dataset.

**Figure 3:**
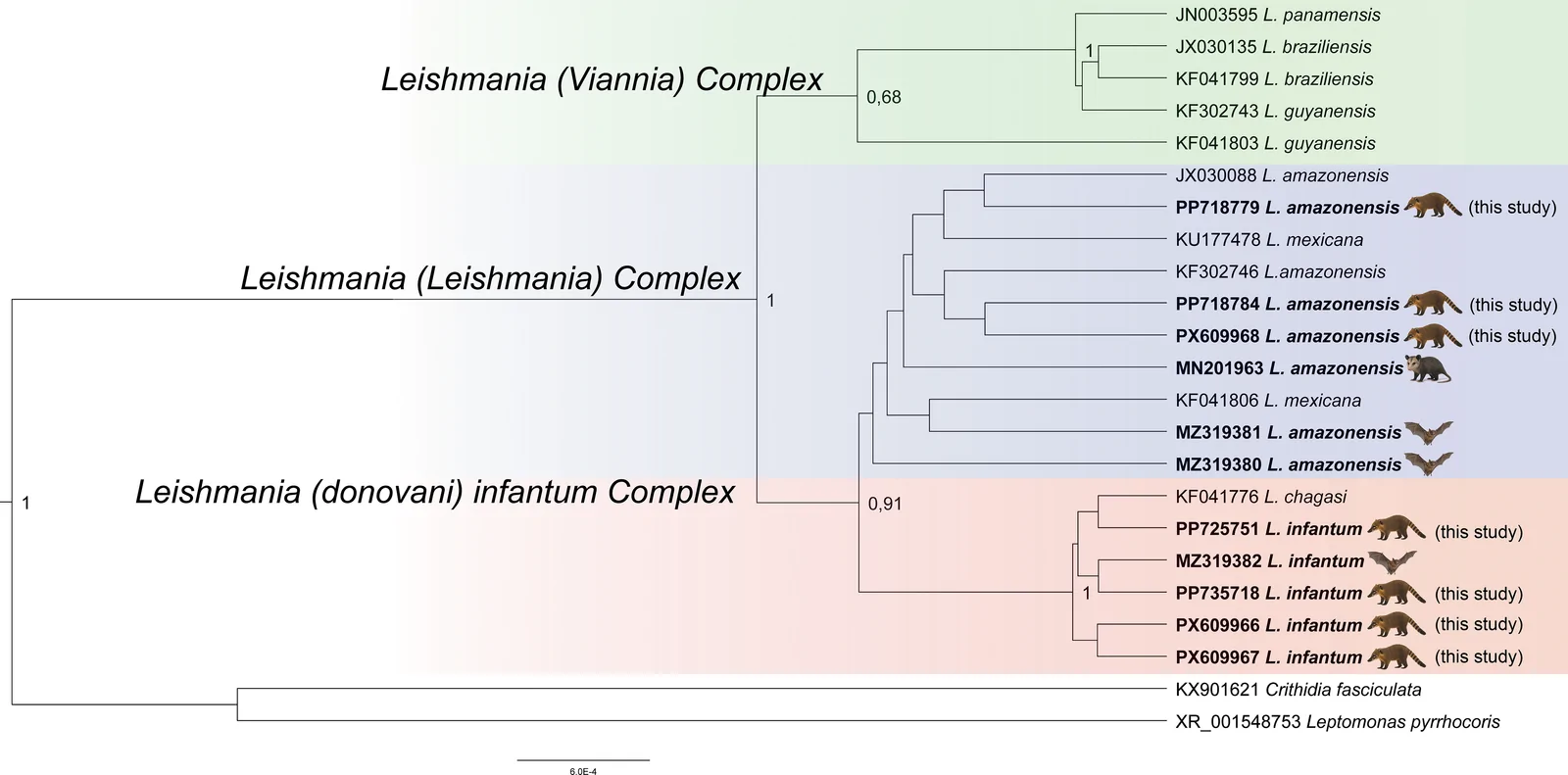
Bayesian inference of *Leishmania* spp. based on the SSU rRNA gene isolated from different host species. The cladogram was inferred using the TrNef substitution model, chosen using the JmodelTest program. The species highlighted in bold are the isolates found in the municipality of Campo Grande - MS. The green rectangle represents the *Leishmania* (*Viannia*) species complex, the blue represents the *Leishmania* (*Leishmania*) complex and the pink represents the *Leishmania* (*donovani*) *infantum* complex. As outgroup, we chose the species *Crithidia fasciculata* (KX901621) and *Leptomonas pyrrhocoris* (XR_001548753).

The second analysis (**Figure 3**), restricted to the genus *Leishmania*, resolved the principal species complexes—*Leishmania (Viannia)*, *Leishmania (Leishmania)*, and the *Leishmania donovani/infantum* complex—placing our isolates of *L. amazonensis* and *L. infantum* in their expected groupings. Owing to the low variability observed among the sequences obtained for *L. infantum* and *L. amazonensis*, a representative subset of sequences from each species was included in the phylogenetic analyses to optimize visualization of the phylogenetic tree. All sequences are listed in **Table 1**.

**Figure 4:**
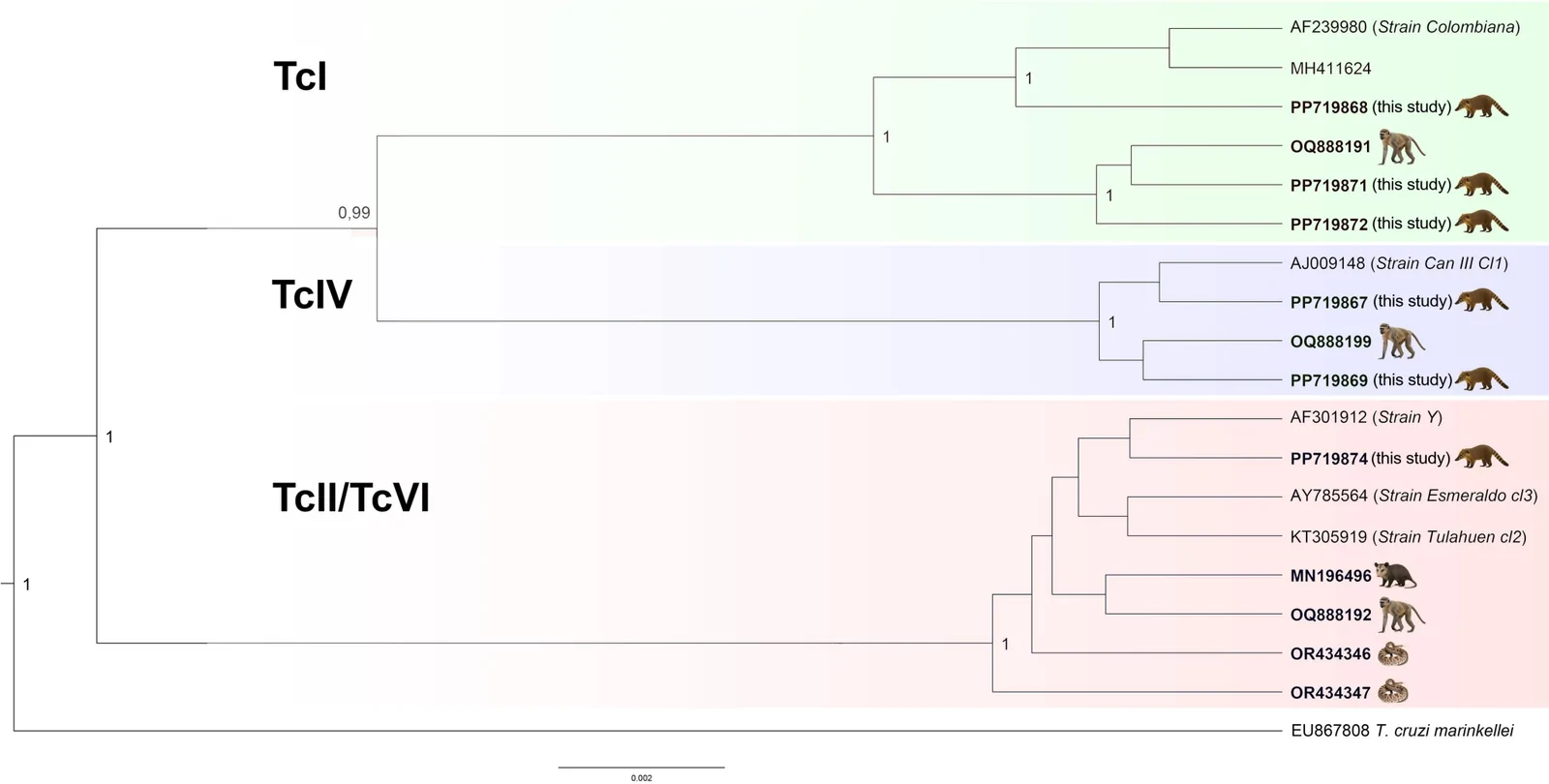
Bayesian inference for the *Trypanosoma cruzi* species, to visualize the Discrete Type Units (DTUs), based on the SSU rRNA gene isolated from hosts. The cladogram was inferred using the K80+I substitution model, chosen using the Jmodeltest program. The specimens highlighted in bold are isolates from Campo Grande - MS. The outgroup used was the *Trypanosoma cruzi marinkellei* species (EU867808). The rectangles highlighted in red, green and blue show clades of *T. cruzi* that are associated with DTUs TcI, Tc IV and TcII/TcVI, respectively.

Finally, we present the putative genotypes of *T. cruzi* (DTUs), enabling direct comparison of samples isolated from hosts in Campo Grande–MS with reference strains that anchor those groupings (e.g., Colombiana, Can III Cl1, Y, Esmeraldo cl3, Tulahuen cl2) and thereby facilitating genotype assignments consistent with the literature (**Figure 4**).

## 4 Discussion

The results obtained in the present study indicate that the coatis living in urban forest fragments of CG in the Brazilian Midwest may act as reservoir hosts for many trypanosomatid species, including those of zoonotic importance as *L. infantum*, *L. amazonensis* and *T. cruzi*. This work (i) reinforces the results obtained by de Macedo et al. (2023) by demonstrating the high occurrence of *L. infantum* in these populations with a different molecular marker (SSU rRNA), (ii) describes for the first time the molecular detection of *L. amazonensis* and Trypanosomatidae sp. CROT in coatis, (iii) exposes a variety of *T. cruzi* DTUs infecting this mammal species in urban environment, and (iv) reveals several types of mixed infections by trypanosomatids in this carnivore species.

The SSU rRNA detection and sequencing results showed that *L. infantum* is more prevalent than other zoonotic trypanosomatid species in coatis from the urban area of CG. Such findings may be explained by the widespread occurrence and overwhelming abundance of *L. longipalpis* over other phlebotomine and triatomine species in the region (Oliveira et al., 2000, 2006; Silva et al., 2007; de Souza Fernandes et al., 2022). Indeed, the adaptive plasticity of *L. longipalpis* to urban environments can be verified in CG over the last few decades: between 1999 and 2000 only 5.7% of captured sand flies corresponded to *L. longipalpis* (Oliveira et al., 2000); between 2004 and 2005, 93.6% of captured sand flies were *L. longipalpis* (Oliveira et al., 2006); the most recent sandfly survey in CG (between 2017 and 2019) revealed that 99.4% of the captured specimen were *L. longipalpis* (de Souza Fernandes et al., 2022). As for triatomines, there is a lack of longitudinal surveys in the urban area of CG, and the only published data on triatomine survey covers 2000 to 2004, indicating a lower rate compared to other municipalities in Mato Grosso do Sul state (de Almeida et al., 2008). This scenario reinforces the endemic characteristic of canine and human VL in CG and certainly explain the high detection rate of *L. infantum* in vertebrate wild hosts as coatis and primates (Júnior et al., 2025) at the region.

In the present study, we report the unprecedent detection of *L. amazonensis* in coatis, confirming the occurrence of other *Leishmania* species than *L. infantum* in coatis from CG, as suggested by de Macedo et al. (2023). In Brazil, *L. amazonensis* is recognized as one of the etiological agents of Cutaneous Leishmaniasis, have a countrywide distribution and is registered in many vertebrate and invertebrate hosts (Brasil et al., 2017; Ratzlaff et al., 2023). In CG, this species has been reported in wild mammal hosts as the Phyllostomidae bats *Carollia perspicillata* and *Platyrrhinus lineatus* (Torres et al., 2024), the non-human primates *Alouatta caraya* and *Sapajus cay* (Júnior et al., 2025), in humans presenting cutaneous leishmaniasis (Souza Castro et al., 2018), and potentially in the opossum *Didelphis albiventris* (Nantes et al., 2021). Our results reinforce the occurrence of *L. amazonensis* in wild mammal species of forested fragments of CG, and may point to the need to perform the differential diagnosis for *L infantum* and *L amazonensis* when testing dogs and humans presenting visceral and cutaneous leishmaniasis, since both clinical presentations have been unusually caused by both *Leishmania* species (Tolezano et al., 2007; Souza Castro et al., 2018). In CG the species determination may be convenient to predict outbreaks by *L. amazonensis*, as well to avoid unneeded euthanasia of symptomatic dogs (Souza et al., 2019).

Our analysis revealed the presence of three *T. cruzi* DTUs in samples of coatis from CG, the TcI, TcIV and TcII/TcVI. Our findings align with other studies examining the phylogenetic diversity of *T. cruzi* in Brazil, indicating a predominance of the TcI genotype. In fact, this genotype is considered the prevalent one in Americas (Brenière et al., 2016). For the remaining genotypes, the clusters were strongly supported, indicating a tendency for the sequences obtained in this study to be grouped with the reference DTUs and with sequences isolated from other wild hosts species in previous studies focusing on urban fragments in the municipality of CG (Nantes et al., 2021; Torres et al., 2024; Júnior et al., 2025). Furthermore, our results reinforce that TcIV and TcII/TcVI circulates in CG in other wild species apart from white-eared opossum, viperid snakes and non-human primates (Nantes et al., 2021, 2024; Júnior et al., 2025).

Interestingly no *T. cruzi* positivity was observed in hemocultures from AFV (Dario et al., 2021) and PEP (this study), indicating low parasitemia at the time of the capture. Similar findings were observed in previous studies with white-eared opossums (Nantes et al., 2021), non-human primates (Júnior et al., 2025) and viperid snakes (Nantes et al., 2024) in the region, where there was substantial prevalence in molecular detection in blood samples, but no positivity in hemocultures. Negative hemocultures together with low occurrence of *T. cruzi* detection in SSU rRNA nPCR (6/110) suggest that coatis, although presenting high populational density (Barreto et al., 2021), are not competent to maintain and disperse *T. cruzi* in CG. This contrast with observations from Pantanal region, where coatis were reported as one of the main reservoirs of *T. cruzi* due to high rates of infection, mainly by TcI and TcII lineages, and high parasitemia (Herrera et al., 2008; Alves et al., 2011; Herrera et al., 2011; Rocha et al., 2013; Alves et al., 2016; Santos et al., 2019). A conceivable hypothesis for this finding is that coatis in the urban environments have a higher availability of food resources (Barreto et al., 2021), resulting in better body condition and nutritional status, and consequent immunological competence to control infection and reduce parasitemia (Malafaia and Talvani, 2011). In fact, evidence of control of *T. cruzi* infection in the two Procyonidae species *Nasua narica* and *Procyon lotor* was presented in a five-year study in a zoological park adjacent to an urban area, where both species presented an infection-reinfection dynamic (Martínez-Hernández et al., 2014), suggesting reduction of parasitemia detectable in PCR at some seasons. Nevertheless, even in low parasitemia, the presence of *T. cruzi* in different wild mammal species living in urban environments, especially those with high populational density, proves relevant when formulating public policies aimed at anticipating future outbreaks in the region.

In the present study, we also detected a Trypanosomatidae that aligned with 100% identity to Trypanosomatidae sp. CROT, detected in rattlesnake (*Crotalus durissus*) from CG (Nantes et al., 2024). This taxon grouped to other trypanosomatid such as Trypanosomatidae sp. ‘strain EVA’ detected in a *Lutzomyia evansi* in Venezuela, and the monoxenous *Sergeia podlipaevi* detected in *Culicoides* spp. from Czech Republic, being suggested by Nantes et al. (2024) as a new independent genus. Our results add knowledge to the Trypanosomatidae ecology field by demonstrating that this possible new genus can be found in mammals in addition to invertebrates and reptile hosts.

In this study, we integrated the results obtained by Dario et al. (2021); de Macedo et al. (2023), and the nPCR of SSU rRNA in the same coati populations. Together, the results revealed infection by at least five trypanosomatid species within the same coati population, including mixed infections by two or three parasite species. A single coati was demonstrated to be coinfected by three different trypanosomatid species within a period of approximately 16 months (*T. cruzi* TcIV, *L. amazonensis* and *C. mellificae*). In this sense, we demonstrate that coatis are capable of bioaccumulate different trypanosomatid species, not only in natural landscapes (Herrera et al., 2008; Rocha et al., 2013) and rural settlements (Porfirio et al., 2018), but also in forested fragments within urban areas. Within urban forested environment, this may be reinforced by the restricted home range that probably favor and boost the interactions between coatis and other hosts species. These interactions may be direct through predation of invertebrates and small vertebrates (Alves-Costa et al., 2004), or indirect through the construction of arboreal nests that may be used by other mammals and invertebrates as a shelter (de Lima et al., 2015; Monticelli and Gasco, 2018; Pessanha et al., 2023).

Previous studies have reported difficulties to characterize trypanosomatid mixed infections in *Rattus rattus* and *D. albiventris* through Sanger sequencing of SSU rRNA, due to the presence of double peak in electropherogram (Dario et al., 2022). In some cases, authors resorted to molecular cloning with pGEM-T Easy Vector System and random selection of colonies to overcome the presence of this technical inconvenience (Rodrigues et al., 2019). In the present study, 72.5% (50/69) of amplified SSU rRNA sequences were characterized through Sanger sequencing with high query cover and identity rate. However, the mixed infections could only be observed in the same individual when using different samples, different techniques, or when exploring samples of different recaptures. These observations demonstrate that Sanger sequencing of SSU rRNA can be a useful alternative in longitudinal studies and in studies that propose the use of more than one type of biological samples and molecular targets.

Our data strongly suggest that monitoring coatis in urban areas should include a longitudinal effort, as well as the study of the blood meal sources of sandflies and reduviid bugs in the urban environment. Furthermore, since arboreal coati nests are used by different species of invertebrate vectors and mammals, they should be included in epidemiological surveillance programs.

## Data Availability

The datasets presented in this study can be found in online repositories. The names of the repository/repositories and accession number(s) can be found in the article/**Supplementary Material**.
